# Eight-and-a-half syndrome: a rare potentially life-threatening disease

**DOI:** 10.3205/oc000086

**Published:** 2018-03-08

**Authors:** Bryan Vincent Q. Mesina, George Michael N. Sosuan, Karen B. Reyes

**Affiliations:** 1 Department of Ophthalmology and Visual Sciences, Sentro Oftalmologico Jose Rizal, University of the Philippines – Philippine General Hospital, Manila, the Philippines

**Keywords:** eight-and-a-half syndrome, cavernoma, neuro-ophthalmology

## Abstract

**Purpose:** To report a case of eight-and-a-half syndrome presenting with a conjugate horizontal gaze palsy, an ipsilateral internuclear ophthalmoplegia, and an ipsilateral lower motor neuron-like facial palsy.

**Methods:** A 56-year-old male who came in for limitation of extraocular muscle motion – wherein the only remaining movement is abduction of the left eye – and right-sided facial weakness. Magnetic resonance imaging revealed a well-defined lesion resembling a cavernoma, at the level of the ponto-medullary junction.

**Results:** Eight-and-a-half syndrome results from a lesion affecting the paramedian pontine reticular formation, the median longitudinal fasciculus, and the facial nerve fascicle on one side. In this case, a cavernoma at the level of the ponto-medullary junction was compressing the important pontine structures.

**Conclusion:** Although eight-and-a-half syndrome is most commonly caused by an infarction or demyelination, in rare instances, a space-occupying lesion at the level of the pons can be the etiology. It is of utmost importance to recognize the features of this disease entity to be able to exhaust the proper diagnostic exams, localize the lesion and determine the proper treatment regimen catered to each patient.

## Introduction

Conjugate horizontal gazes are binocular movements that move the eyes synchronously toward one direction of gaze. These movements are supranuclear in origin, control arising from the cerebrum and brainstem. The primary horizontal gaze control center is at the paramedian pontine reticular formation (PPRF) located at the pons, at the level of the facial colliculus. The PPRF sends signals towards its ipsilateral abducens nerve (VI) and contralateral medial longitudinal fasciculus. A lesion of this region would result to horizontal conjugate gaze palsy [1], [2].

Internuclear ophthalmoplegia (INO) is a disorder where there is defective adduction of the eye on the affected side and ataxic horizontal jerky nystagmus of the contralateral eye on abduction. Gaze towards the side of the lesion is, however, normal. INO arises from a lesion of the medial longitudinal fasciculus (MLF), a pair of crossed axonal tracts situated near the brainstem. It receives signals from the contralateral PPRF and in turn sends signals to its ipsilateral oculomotor nerve, coordinating conjugate eye movements. Lesions leading to INO include stroke, space-occupying lesions and demyelination [1], [2].

Lesions affecting both the PPRF and MLF on one side lead to the formation of the neuro-ophthalmologic disease entity one-and-a-half syndrome. First described by Fisher, one-and-a-half syndrome is a disorder comprised of ipsilateral conjugate horizontal gaze palsy, known as the “one” and ipsilateral internuclear ophthalmoplegia (INO), known as the “half”. The only remaining ocular movement is abduction of the contralateral eye [2]. In rare occasions, a lesion may affect the PPRF, MLF, and its ipsilateral facial nerve fascicle around the area of the facial colliculus as it goes around the abducens nucleus. This lesion may give rise to a one-and-a-half syndrome with associated ipsilateral facial muscle weakness, also known as eight-and-a-half syndrome.

Eight-and-a-half syndrome is a rare pontine neuro-ophthalmologic syndrome made up of a conjugate horizontal gaze palsy, ipsilateral internuclear ophthalmoplegia and ipsilateral lower motor neuron-like facial palsy [1], [3], [4], [5], [6], [7]. The facial palsy may involve forehead weakness, orbicularis weakness, facial asymmetry, and shallow nasolabial fold [1]. It is most often caused by a vascular etiology as in an infarction, as first described by Eggenberger in 1998 when magnetic resonance angiography demonstrated eight-and-a-half syndrome caused by vertebral basilar disease [8]. Kakar et al., Sampath Kumar et al., Nandhagopal et al., and Bocos-Portillo et al. also demonstrated eight-and-a-half syndrome as a sequelae of pontine infarction as seen in magnetic resonance imaging [3], [4], [5], [9].

Eight-and-a-half syndrome can also be caused by demyelinating conditions at the level of the pons, such as multiple sclerosis, as demonstrated by Skaat et al. and Mortzos et al. [2], [10]. In even rarer instances, eight-and-a-half syndrome can be caused by a space-occupying lesion at the level of the pons such as a tuberculoma, as demonstrated by Van Toom and colleagues [11].

Since a majority of the cases is secondary to infarction or ischemia at the pontine level, its onset is often sudden [3]. No sexual, race or age group predilection has been noted since the disease has first been documented. Hypertension, diabetes mellitus, and hypercholesterolemia are the co-morbidities noted commonly amongst patients with the disease [3], [5], [6], [9]. Prognosis of cases involving infarcts depends on the ability of the affected area to recover [4], [5], [9]. Several studies note improvement of the facial palsy before other signs and symptoms [4]. Neuro-rehabilitation is mostly warranted [3]. Prognosis of cases due to demyelination depends on the progression of the primary disease entity [2], [10]. In cases dealing with space-occupying lesions, recovery depends on the removal of the lesion, as demonstrated by Van Toom and colleagues [11].

## Case description

A fifty-six-year-old hypertensive male came in for consult due to an inability to abduct the right eye. History of the present illness started about thirty-four years prior when he first noted a limited abduction of the right eye. During the interim, he noted a gradually progressive limitation in all movements of the right eye, and a limitation in adduction of the left eye, associated with occasional diplopia. No consults were sought until a month prior when he noticed a sudden-onset right-sided facial weakness and dysarthria, with no hemiparesis or hemiplegia.

The patient came into the clinic with good visual acuity, good color vision, and no relative afferent pupillary defect, but with a right horizontal conjugate gaze palsy, a limitation of adduction of the right eye with associated nystagmus of the left eye on abduction, and a right peripheral facial nerve palsy demonstrated by a four-millimeter lagophthalmos, a shallow nasolabial fold, and an inability to wrinkle the forehead. The rest of the neurologic examination was unremarkable. Slit lamp examination was also essentially unremarkable except for immature cataracts. Indirect ophthalmoscopy showed evidence of hypertensive retinopathy on both eyes.

**Figure 1:**
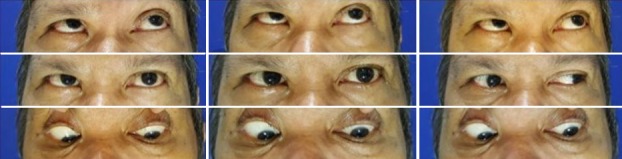
Composite diagram

Figure 1 (Fig. 1) shows the patient’s extraocular muscle movements in a composite diagram. There is a right horizontal gaze palsy and a limited adduction of the right eye. The sole extraocular muscle movement remaining is an abduction of the left eye.

**Figure 2:**
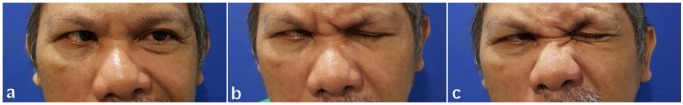
a) Shallow nasolabial fold, right, b) lagophthalmos, right, c) corrugator supercilli and procerus muscle weakness

Figure 2 (Fig. 2) highlights the peripheral facial nerve palsy. The patient has a shallow nasolabial fold on the right, a four-millimeter lagophthalmos on the right, and weakness of the occipitofrontalis, corrugator supercilli and procerus muscles.

**Figure 3:**
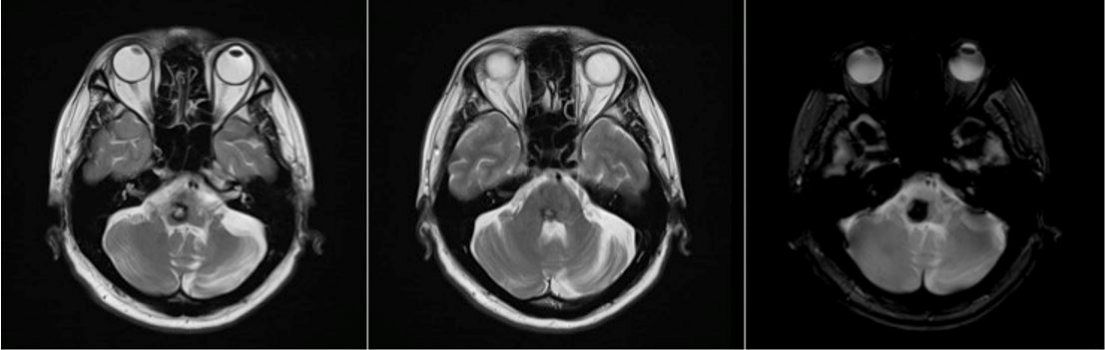
T2-weighted images from the patient’s cranial magnetic resonance imaging

**Figure 4:**
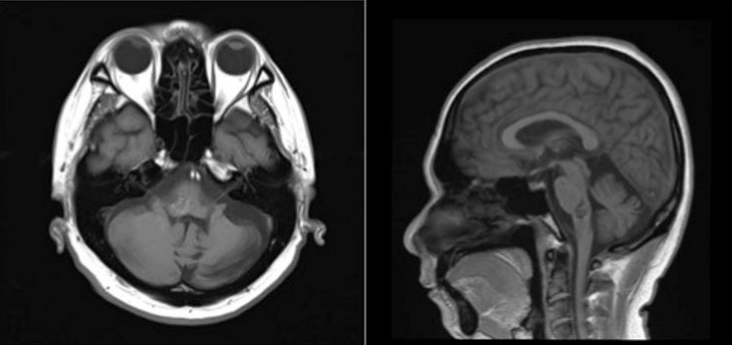
T1-weighted images from the patient’s cranial magnetic resonance imaging

**Figure 5:**
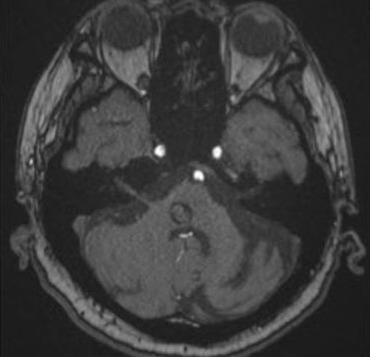
Time of flight image from the patient’s cranial magnetic resonance angiogram

On magnetic resonance imaging, there is a hemorrhage with a complete hemosiderin ring at the level of the pons/ponto-medullary junction, associated with a well-defined lesion with a “popcorn ball” appearance resembling that of a cavernoma (Figure 3 (Fig. 3), Figure 4 (Fig. 4), and Figure 5 (Fig. 5)).

The patient was co-managed with neurosurgery and neurology services for possible resection.

## Discussion

Presented above is the case of a middle-aged male with a horizontal gaze palsy, an ipsilateral internuclear ophthalmoplegia, and an ipsilateral peripheral facial nerve palsy, with no other associated findings. On imaging, a cavernoma with surrounding hemorrhage is noted at the level of the pons. Anatomically, the area in which the lesion is situated in correlates well with the features seen in the patient’s case. Cavernomas, also known as cerebral cavernous malformations, are common vascular malformations in the brain, accounting to 10–25% of all vascular malformations [12]. The incidence in the general population is 0.4–0.8%, and the age group in which most are first diagnosed is within the twenty to forty year-olds [12]. 

Cavernomas are composed of clusters of abnormal, hyalinized capillaries surrounded by hemosiderin deposits and gliotic reaction [12]. They can be found in any location in the brain, but 70–80% are supratentorial in origin [12]. Supratentorial cavernomas present with new-onset seizures and headaches while infratentorial cavernomas present with progressive neurologic deficits. Due to their slow-growing but dynamic nature, patients with cavernomas may remain asymptomatic or may only have focal neurologic deficits. However, sudden-onset, neurologic deficits occur in cases of acute hemorrhage. If left untreated, cavernomas may lead to intracerebral hemorrhage, seizures, focal neurologic deficits, or headaches of varying severity [12]. The annual risk of hemorrhage is 0.7–1.1% per lesion in patients with no history of hemorrhage, but rises to 4.5% in patients with a previous episode of intracerebral hemorrhage [12]. Going by location, there is a 3.8% risk of hemorrhage in infratentorial cavernomas, compared to a 0.4% risk in supratentorial cavernomas [12]. 

In this case, symptoms started more than thirty years ago, which may have been the exact time when the lesion started to form. As the lesion grew to its present size, the symptoms worsened. Eventually, the cavernoma may have then ruptured leading to a hemorrhage, which may have encroached up to the level of the facial colliculus leading to the constellation of signs and symptoms in this patient, presenting as eight-and-a-half syndrome.

Cavernomas are angiographically occult lesions, which is why conventional T1- and T2-weighted magnetic resonance imaging (MRI) is still being used for its diagnosis [12]. The MRI is able to accurately detect cavernomas, which have a pathognomonic “popcorn-ball” appearance due to loculated hemorrhages, hemosiderin deposition around the primary lesion, and gliotic reaction [12]. Based on imaging, differentials may include hemorrhagic brain metastasis, gliomas, and hemorrhagic telangiectasias (Osler-Weber-Rendu disease) [12].

Microsurgical resection via the trans-sulcal approach, stereotactic radiosurgery (gamma knife surgery), and conservative management are the treatment options [12], which is why the patient was referred to the neurosurgery service. The choice of management depends on a myriad of factors including accessibility and location of the lesion, size of the lesion, severity of clinical presentation, and patient preference.

However, presented with the risks and benefits of the surgical procedure, the patient and his family opted to choose conservative management and have the disease run its course. In conservative management, alleviation of clinical symptoms is the primary goal. Pain relievers and anti-seizure medications may be prescribed and patients are advised to refrain from strenuous exercises, anticoagulant use and pregnancy [12].

The area encompassed by the structures affected in eight-and-a-half syndrome spans a large portion of the pons. Its presentation can mimic a cerebrovascular accident, but an affected area that large should irk an ophthalmologist to consider other etiologies, such as space-occupying lesions. Hence, urgent and immediate localization of the lesion via radiologic examinations is of utmost importance.

## Conclusions

Conjugate horizontal gaze palsy, ipsilateral internuclear ophthalmoplegia, and ipsilateral peripheral facial nerve palsy can occur together as a constellation of features seen in eight-and-half syndrome. Although it is most commonly caused by an infarction or demyelination, in rare instances, a space-occupying lesion, such as a cavernoma located at the level of the pons, can be the etiology. It is important to recognize the features of the disease so as to be able to exhaust the proper diagnostic exams, localize the lesion, and determine the proper treatment regimen catered to each patient.

## Notes

### Competing interests

The authors declare that they have no competing interests.

### Literature search

PubMed was searched for English-language articles on May 10, 2017, using the following terms: *Eight-and-a-Half Syndrome*, *Cavernoma*, *Neuro-Ophthalmology*. Sources in retrieved articles were cross-referenced.

### Acknowledgments

The authors thank the Neuro-Ophthalmology Service of the University of the Philippines-Philippine General Hospital Department of Ophthalmology and Visual Sciences for the support and guidance in making this paper.

### Ethical consideration

The case report is a minimal risk study which was conducted in full compliance with the principles of the 7^th^ iteration of the Declaration of Helsinki and Good Clinical Practice of the WHO. All identifying patient information was kept confidential. Informed consent from the patient was obtained prior to inclusion in the study.
